# The active core microbiota of two high-yielding laying hen breeds fed with different levels of calcium and phosphorus

**DOI:** 10.3389/fphys.2022.951350

**Published:** 2022-09-23

**Authors:** Christoph Roth, Tanja Sims, Markus Rodehutscord, Jana Seifert, Amélia Camarinha-Silva

**Affiliations:** ^1^ HoLMiR—Hohenheim Center for Livestock Microbiome Research, University of Hohenheim, Stuttgart, Germany; ^2^ Institute of Animal Science, University of Hohenheim, Stuttgart, Germany

**Keywords:** laying hens, core intestinal microbiota, functional prediction, active community, dietary treatment

## Abstract

The nutrient availability and supplementation of dietary phosphorus (P) and calcium (Ca) in avian feed, especially in laying hens, plays a vital role in phytase degradation and mineral utilization during the laying phase. The required concentration of P and Ca peaks during the laying phase, and the direct interaction between Ca and P concentration shrinks the availability of both supplements in the feed. Our goal was to characterize the active microbiota of the entire gastrointestinal tract (GIT) (crop, gizzard, duodenum, ileum, caeca), including digesta- and mucosa-associated communities of two contrasting high-yielding breeds of laying hens (Lohmann Brown Classic, LB; Lohmann LSL-Classic, LSL) under different P and Ca supplementation levels. Statistical significances were observed for breed, GIT section, Ca, and the interaction of GIT section x breed, P x Ca, Ca x breed and P x Ca x breed (*p* < 0.05). A core microbiota of five species was detected in more than 97% of all samples. They were represented by an uncl. *Lactobacillus* (average relative abundance (av. abu.) 12.1%), *Lactobacillus helveticus* (av. abu. 10.8%), *Megamonas funiformis* (av. abu. 6.8%), *Ligilactobacillus salivarius* (av. abu. 4.5%), and an uncl. *Fusicatenibacter* (av. abu. 1.1%). Our findings indicated that Ca and P supplementation levels 20% below the recommendation have a minor effect on the microbiota compared to the strong impact of the bird’s genetic background. Moreover, a core active microbiota across the GIT of two high-yielding laying hen breeds was revealed for the first time.

## Introduction

The laying hen gastrointestinal tract (GIT) microbiota consists of a complex community of diverse microorganisms. The host influences the composition of the microbial community, which may have effects on the immune system, nutrient digestion, and regulation of intestinal physiology (Stanley et al., 2014; Agus et al., 2018; Khan et al., 2020). Depending on the diet and nutrient supplementation, variations in microbial composition can be observed (Leeming et al., 2021). Moreover, it is essential to understand the inter-relation between diet, microbiota, and host when investigating how they contribute to animal health.

Diets are formulated to fulfil the needs of the animals, and the specifically required nutrient concentrations are dependent on the host age, physiological status, and level of performance. Among required minerals, phosphorus (P) and calcium (Ca) are vital because of their function in avian biochemical pathways and bone and eggshell development (Selle et al., 2009). However, P supplements are costly and negatively impact the environment when accumulated in the excreta of the animals. This has stimulated research on hydrolysis of phytate, which is the main binding form of P in plants, in poultry’s digestive tract and variation in the level of P supplementation (Rodehutscord et al., 2022). The influence of age, genotype and experimental design variations affect the results’ comparability (Kebreab et al., 2009; Ahmadi and Rodehutscord, 2012; Deusch et al., 2015; Forgie et al., 2019). The Ca concentration of the feed is related to P, and in laying hens, the highest Ca requirement is during the laying period (Kebreab et al., 2009; Ahmadi and Rodehutscord, 2012). In this phase, the animal requirements must be fulfilled to maintain animal health and performance. Digested and undigested dietary compounds influence the microbial population in the GIT, which modifies the host intestinal integrity and improves pathogen resistance (Forgie et al., 2019). Moreover, there is a microbial distinction between mucosa and digesta samples (Deusch et al., 2015; Waite and Taylor, 2015). Mucosa samples of the gastrointestinal tract have shown higher microbial diversity than digesta samples (Borda-Molina et al., 2016). The complex microbial diversity in both sample types consists of hundreds of species across different phyla, inhibiting a clear understanding of GIT variations (Borda-Molina et al., 2016).

Little is known about the dynamics and influence of common active bacteria on the GIT of laying hens. Therefore, the microbiota’s response to a specific challenge and environment by targeting the active community has to be reflected. Despite showing similar diversity to total communities, the microbial taxa composition is significantly different (Bastida et al., 2017). Shade and Handelsman (2012) defined that the core microbiome consists of shared microbial members within similar habitats and across complex microbial assemblages. Furthermore, a core microbiome is present and interacts in the entire GIT. In addition, transient or resident bacteria can be considered a core microbiome. It is an approach to understanding, adjusting, and optimizing microbial functions in individuals or complete ecosystems (Heumann-Kiesler et al., 2021; Hofmann et al., 2021). Knowledge about microbial changes across different GIT sections can help understand specific processes, e.g., food fermentation or predicting and controlling the microbiome (Giraffa, 2004; Stegen et al., 2018; Berg et al., 2020).

This study aimed to evaluate the impact of different concentrations of P and Ca on the active microbiota of the GIT (crop, gizzard, duodenum, ileum, caeca) of two high-yielding laying hen breeds and determine how the host genetic background and dietary changes influence the resident core microbiota.

## Materials and methods

### Sample collection, DNA extraction, and illumina library preparation

This research complements and extends recent publications (Sommerfeld et al., 2020; Heumann-Kiesler et al., 2021; Hofmann et al., 2021). Samples originated from an animal trial fully described by Sommerfeld et al. (2020). The study was approved by the Regierungspräsidium Tübingen (approval number HOH50/17 TE) and conducted following animal welfare regulations. Animals were housed at the University’s Agricultural Experimental Station (Unterer Lindenhof, Eningen, Germany).

A total of 80 laying hens of the breeds Lohmann brown-classic (LB) and Lohmann LSL-classic (LSL) were used in this study. Upon the arrival of the hatchlings at the farm, birds were raised together under the same conditions (floor pens, deep litter bedding on wood shavings, and diets). At 27 weeks, ten hens per breed were allocated to four dietary treatments in a randomized design and kept individually in metabolism units. The individuals received water and feed for ad libitum consumption for 3 weeks. Soybean meal and corn-based diets were supplemented to reach a standard (5.3 g/kg dry matter (DM); P^+^) or reduced (4.7 g/kg DM; P^-^) P concentration and a standard (39.6 g/kg DM; Ca^+^) or reduced (33.9 g/kg DM; Ca^-^) Ca concentration. Diets ingredient compositions are fully described in Sommerfeld et al. (2020).

At 31 weeks of life, birds were stunned with a gas mixture of 35% CO_2_, 35% N_2_, and 30% O_2_ and sacrificed by decapitation. The crop (Cr), gizzard (G), duodenum (D), ileum (I) and caeca (Cae) were longitudinally opened, digesta was obtained with a sterile spoon, and after a cleaning step with sterile phosphate-buffered saline solution, the mucosa was collected by scratching it with a sterile glass slide. Collected samples were immediately stored in RNA later at −80°C until further analysis. RNA of a total of 800 samples were extracted using Trizol (Invitrogen Inc., Waltham, United States) according to the manufacturer’s instructions with a preliminary step of bead beating (30 s, 5.5 m/s) in a FastPrep instrument (MP Biomedicals, Eschwege, Germany). RNA was quantified with Nanodrop (ThermoFisher Scientific, Waltham, United States) and stored at −80°C until further analysis. RNA samples were treated with the DNase kit (Invitrogen), and cDNA synthesis was performed using SuperScript III First-Strand Synthesis System for RT-PCR (Invitrogen).

Sequencing libraries were made according to the protocol described by Borda-Molina et al. (2020). All PCR reactions were done with PrimeSTAR^®^ HS DNA Polymerase kit (TaKaRa, Beijing, China). The first two PCR were prepared in a total volume of 25 µl using 1 µl of cDNA template, 0.2 µM of each primer and 0.5 U Taq prime start HS DNA and the third PCR was set up in a total volume of 50 µl. An initial denaturation at 95°C for 3 min was followed by ten cycles (first and second PCR) or 20 cycles (third PCR) of denaturation at 98°C for 10 s, annealing at 55°C for 10 s and an extension at 72°C for 45 s and a final extension of 72°C for 2 min. PCR products were purified and standardized using SequalPrep Normalization Kit (Invitrogen Inc., Waltham, United States) and sequenced using 250 bp paired-end sequencing chemistry on Illumina Novaseq 6000.

### Bioinformatics and statistical analysis

The bioinformatic analysis was performed with Mothur v1.44.3 (Schloss et al., 2009). Raw reads (forward and reverse fastq file) were assembled with make.contigs function. Reads with ambiguous bases, with homopolymers (>8) and longer than 354 bp were removed. A total of 678 samples passed this filtering and were used for downstream-analysis. Sequences were aligned to the silva.seed v1.38.1 (Quast et al., 2013). Chimeras were identified using vsearch (Rognes et al., 2016) and removed from the dataset. Sequences were classified using the Bayesian classifier and the Silva reference and taxonomy set silva.seed v1.38.1. The output was filtered to get the amplicon sequencing variants (ASVs) with a minimum of 50 reads across all samples resulting in 6179 ASVs. An average of 34.566 ± 17.567 reads were obtained per sample. The cut-off for bacterial taxonomy classification followed the recommendations of Yarza et al. (2014). Digesta and mucosa samples have been merged for further analysis per section and considered gastrointestinal tract sections. Sample reads were standardized, and a sample-similarity matrix based on the Bray-Curtis similarity coefficient (Bray and Curtis, 1957) was created using Primer6 (Clarke and Warwick, 2001). PERMANOVA routine was used to study the significant differences and interactions between groups and diets (Clarke and Warwick, 2001). Steel-Dwass test was performed to compare means of relative abundance data between genera and breed (Br), gastrointestinal tract section (GS), and Ca/P level combinations using JMP®Pro (Version 16.1 SAS Institute Inc., Cary, NC, 1989–2021). P-values based on ANOSIM results were adjusted using the Benjamin-Hochberg correction (FDR). The core microbiota across all samples was identified with the phyloseq and microbiome library in R v4.1 (McMurdie and Holmes, 2013; Lahti et al., 2017). ASV table, taxonomy information, and metadata were combined in a phyloseq file. Groups were subset according to the metadata (diet, GS and Br) to create a phyloseq file for each combination of the three factors. All phyloseq files of all groups were standardized by ASVs. The detection level of core members was set to 0.01% of abundance and a prevalence of 97% across all samples. The output ASV list was compared between all groups to determine the common ASVs, and venn diagrams were drawn with the InteractiVenn tool (Heberle et al., 2015).

The Shannon diversity index and richness were calculated using the phyloseq library in R v4.1. LDA scores were analyzed with microbiomeAnalyst (Chong et al., 2020). Data filter and normalization were set to default. P-values threshold was set to *p* = 0.05 and the FDR correction was applied. LEfSe-graphs were built with the build-in graph builder (Segata et al., 2011).

Functional prediction was performed in R with the latest version of Tax4Fun2 v1.1.5 (https://github.com/bwemheu/Tax4Fun2). Bacterial genomes detected on the microbiota dataset were downloaded from the NCBI database, and a reference database was created to improve functional accuracy. Functional predictions were then performed using the reference file and the ASV table of all samples. The threshold for clustering (uclast) was set to 100%, and the number of 16S rRNA copies were normalized and calculated for each ASV.

## Results

### Experiment evaluation

**FIGURE 1:**
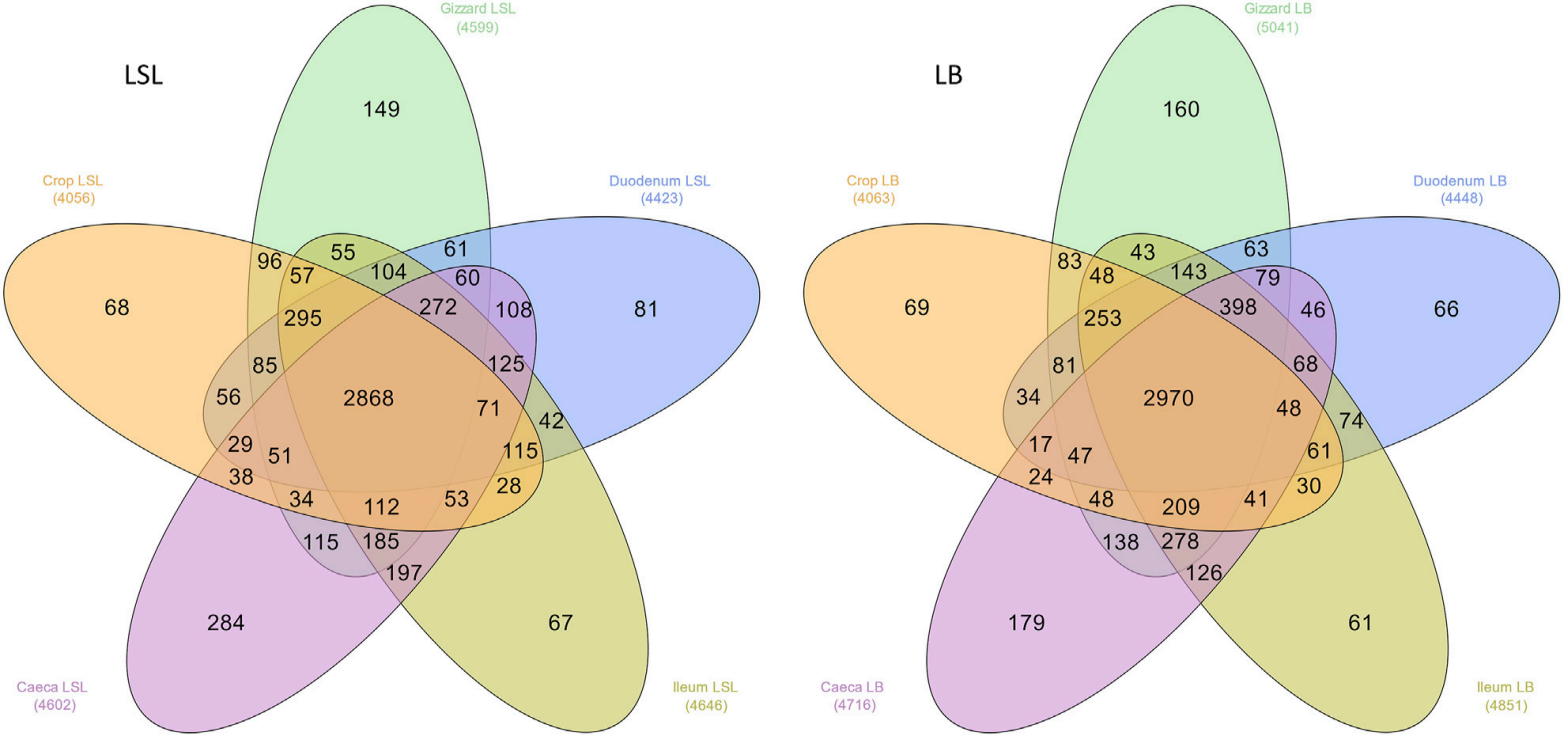
Distribution of the total number of ASVs among GIT sections across all samples in both breeds. The number in parenthesis is the observed number of ASVs in each group.

The overall microbiota consisted of 6179 ASVs, where 2272 ASVs were shared by all GIT sections, breeds, and dietary treatments. LSL samples shared 2868 and the LB 2970 (Figure 1). The number of unique ASVs varied from 61 to 284, depending on the breed and GIT section. Moreover, the breed comparison of each GIT section revealed that many ASVs were unique for each breed (Supplementary Figure S1).

According to the sequencing data, the microbiota of all samples consisted of Firmicutes (average relative abundance [av. abu]) of 84.5% in LSL and 76.7% in LB (*p* < 0.05), followed by Bacteriodetes, which was more abundant in LB (18.2%) in comparison to LSL (10.7%) (*p* < 0.05) (Supplementary Figure S2A). The most abundant genera were *Lactobacillus* (25.1% LSL; 17.4% LB), followed by uncl. Lactobacillaceae (21.2% LSL, 8.2% LB), uncl. Lachnospiraceae (10.8% LSL, 13.5% LB), and *Ligilactobacillus* (7.9% LSL, 12.5% LB). These genera reached an average relative abundance of more than 50% across all samples (Supplementary Figure S2B). Additionally, significant differences were found between breeds and GIT sections within the breeds (Supplementary Table S1).

**FIGURE 2:**
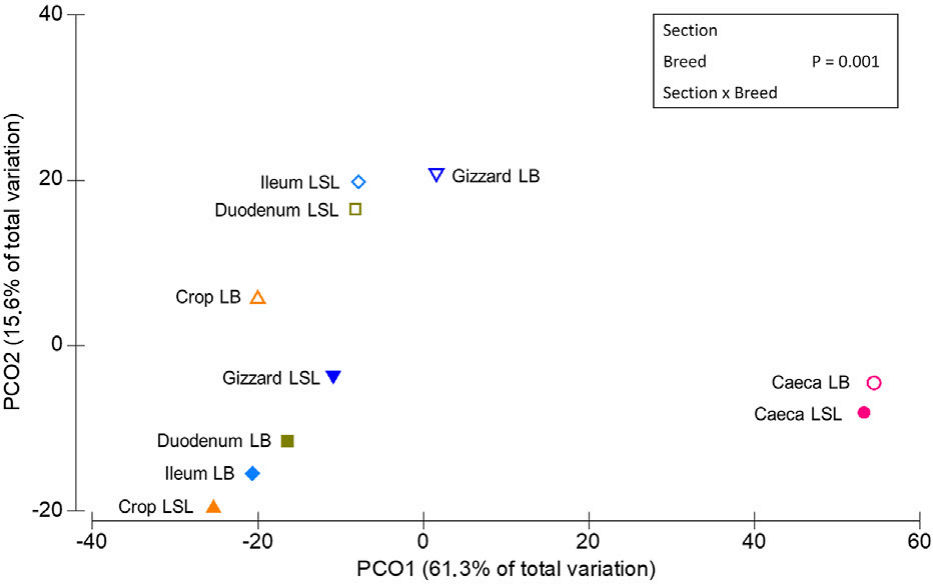
Multidimensional scaling of centroids showing the similarities among the sample types derived from sample combinations of GIT section x breed.

PERMANOVA routine was used to study the overall significant differences and interactions between GIT sections, laying hen breeds, P and Ca supplementation. A statistical significance on ASV level was reached for each factor alone (*p* < 0.03) and the interactions between Br x GS, Br x Ca, P x Ca, P x Ca x Br (*p* < 0.03). A trend was observed for Br x P (*p* = 0.09) (Supplementary Table S2). The principal coordinates analysis plot revealed three clusters (Figure 2), one comprising the LSL samples of crop, gizzard, duodenum and ileum, another with those same samples but for the LB breed and a third one with the caeca samples of both breeds.

In crop samples, significant effects of the breed and Ca and a trend for the interactions of Br x Ca (*p* < 0.08) were observed. The gizzard, duodenum and ileum microbiota were significantly affected by the breed (*p* < 0.05). In the caeca, significant effects of the breed, P/Ca supplementation, the interactions of Br x Ca, Ca x Br, P x Ca x Br (*p* < 0.03) and a trend for P x Br were detected (*p* < 0.08). All significant interactions are provided in Supplementary Table S2.

Pairwise comparisons evaluating the Ca and P supplementation effects on the breed and GIT section, exhibited significant effects, depending on the GIT section. For an overview, see Supplementary Table S3. A significant difference was detected regarding P supplementation for LB caeca P^+^ vs. P^-^ (*p* < 0.01). An effect of the Ca supplementation was observed in both breeds. In LB, a significant difference was identified in crop Ca^+^ vs Ca^-^ (*p* = 0.02) and caeca Ca + vs Ca- (*p* < 0.01) was revealed. For LSL, significant differences were observed in caeca Ca^+^ vs Ca^-^ (*p* < 0.01). However, the strongest effect was driven by the breed rather than GIT section, Ca or P supplementation levels. The breed effect is clearly shown in caeca samples (Supplementary Figure S3), and all significant *p*-values are shown in Supplementary Table S3.

**FIGURE 3:**
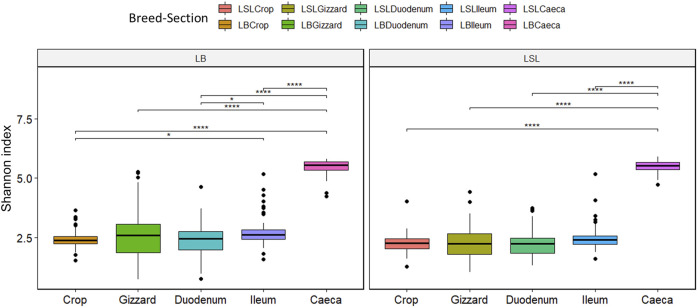
Boxplot of Shannon diversity index separated by the breed, section (color) and Ca/P combination of the diet (*p < 0.02; ****p < 0.001).

The LB showed significantly higher overall Shannon diversity (3.09) than LSL (2.93). A statistical significance between caeca and all GIT sections was observed for both breeds (*p* < 0.05). For the LB additional significances were observed between ileum and crop and ileum and duodenum. (*p* < 0.03) (Figure 3). Regarding the diet, the Shannon index differed depending on the GIT section and breed combination. Still, no statistical significance was observed between diets, with the highest index observed in caeca (Supplementary Figure S4).

### Functional prediction

A total of 322 pathways and 7516 functions were assigned to the samples. Thirty KEGG pathways contributed to more than 50% of the total pathways across all samples and revealed significant differences between breeds and/or GIT sections of the same breed. These thirty KEGG pathways belonged to twelve second-level KEGG functional categories. The global/overview metabolism map was the most enriched function, followed by membrane transport metabolism and signal transduction. Significant effects in the caeca were observed for the breed and the interaction of Br x P (*p* < 0.05) (Supplementary Table S4). Two of the top 30 pathways [ko02010 (ABC transporters) and ko00190 (oxidative phosphorylation)] showed significant breed effects (*p* < 0.05). Despite the significance of breed × P interaction, only one inositol related individual function [K06607 (myo-inositol catabolism protein IolS)] showed differences in LSL (Supplementary Table S4). Regarding Ca supplementation and its effect on the caeca, a significant difference was detected for the myo-inositol catabolism protein IolS (K06607, *p* = 0.01) in LSL, and scyllo-inositol 2-dehydrogenase (NADP^+^) (K22230, *p* < 0.05) in LSL and LB. In addition five other inositol related functions show breed effects (Supplementary Table S4).

### Core Microbiota

**FIGURE 4:**
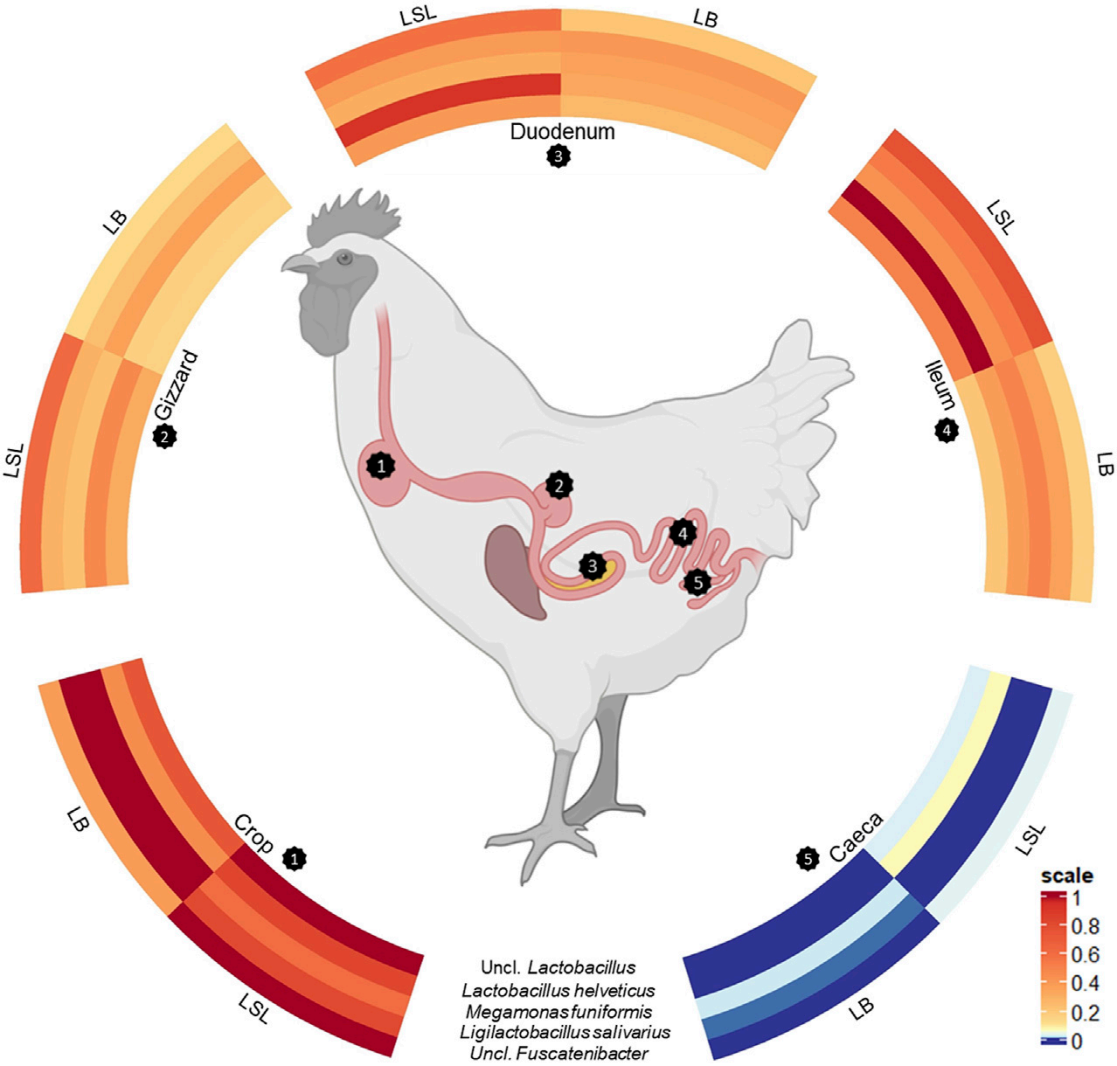
Scaled circulized heatmap of the five core microbiota separated by the GIT sections (crop, gizzard, duodenum, ileum, and caeca) and breed (LSL, LB).

A total of five ASVs were present in 97% of all samples (Figure 4). The core microbiota was represented by an uncl. *Lactobacillus* (ASV62, av. abu. 12.1%), *Megamonas funiformis* (ASV63, av. abu. 6.8%), *Ligilactobacillus salivarius* (ASV 137, av. abu. 4.5%), *Lactobacillus helveticus* (ASV197, av. abu. 10.8%) and uncl. *Fusicatenibacter* (ASV 561, av. abu. 1.1%). Except for the gizzard of LB and caeca of both breeds, the five bacteria accounted for 25%–71% of the total community (Supplementary Table S5). Uncl. *Lactobacillus* was more abundant in LSL compared to LB in all GIT sections (Supplementary Table S5). The highest abundance of *Megamonas funiformis* (ASV63) was observed in the crop of both breeds (Supplementary Table S5). *Ligilactobacillus salivarius* (ASV137) had the highest abundance in the crop and the lowest in the caeca. Furthermore, it was present in higher abundance in LB than LSL (Supplementary Table S5). Also, significant differences were shown between breeds in crop and between GIT sections within the breeds (*p* < 0.05, Supplementary Table S5). *Lactobacillus helveticus* (ASV197) was more abundant in all GIT sections of LSL, with the highest average relative abundance in the ileum, followed by duodenum and crop (Supplementary Table S5). Additionally, significant differences between breeds in all GIT sections (*p* < 0.05, Supplementary Table S5). Uncl. *Fusicatenibacter* (ASV561) was detected in very low abundances across the gastrointestinal tract (Supplementary Table S5). Moreover, significant differences existed between breeds and GIT sections within the breeds (*p* < 0.05, Supplementary Table S5).

### The effect of P and Ca supplementation on the genera distribution and the core microbiome across the gastrointestinal tract

The Ca supplementation affected the microbial composition in LB crop (*p* < 0.05), and significant effects were found for the genus uncl. Lactobacillaceae and *Streptococcus* (*p* < 0.01) (Supplementary Figure S5). Further, the average relative abundance of uncl. Lactobacillaceae increased while *Streptococcus* decreased with Ca supplementation in the diet. Despite the higher diversity of the caeca, fewer differences at genus level were observed for Ca supplementation. Significant changes in LSL were observed for uncl. Bacteroides, uncl. Lachnospiraceae, *Ligilactobacillus* and *Megasphaera* in LB (*p* < 0.10) (Supplementary Figure S5). The average abundance of all genera increased by supplementing Ca except for uncl. Lachnospiraceae.

Significant shifts in the genera *Helicobacter,* uncl. Gammaproteobacteria, and uncl. Prevotellaceae and the trends for *Lachnoclostridium* and *Megasphaera* supported the significant P effect in LB caeca (Supplementary Figure S5). In addition, P supplementation increased the average abundance of uncl. Prevotellaceae, *Helicobacter,* and *Lachnoclostridium* while decreasing *Megasphaera* and uncl. Gammaproteobacteria.

**FIGURE 5:**
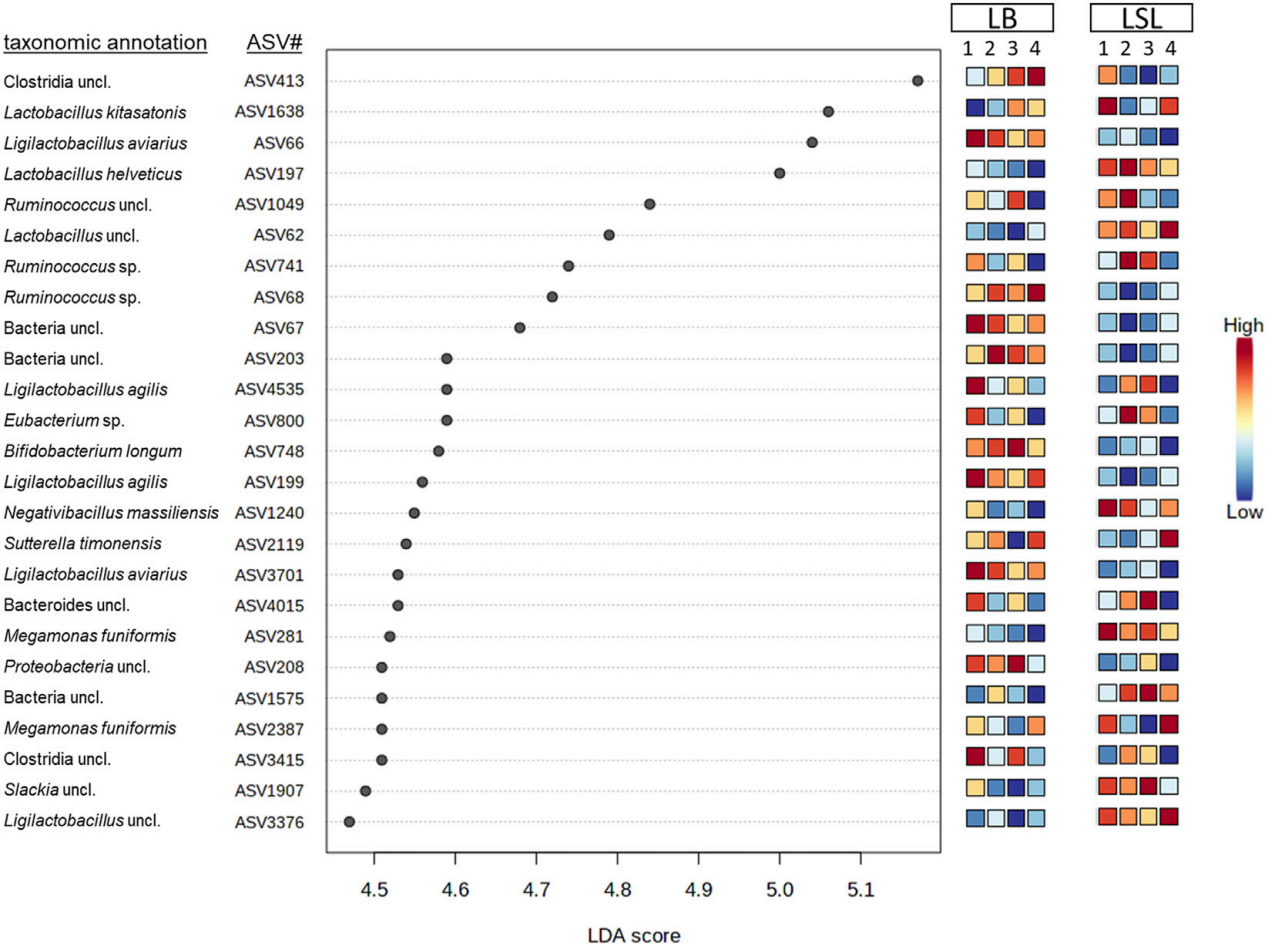
Discriminant analyses of the 25 most significant ASVs in caecal samples based on a LEfSe analysis showing the impact per diet (1: P^+^Ca^+^, 2: P^-^Ca^-^, 3: P^+^Ca^-^, 4: P^-^Ca^+^) and breed. The scale indicates the relative abundance in comparison to the average across the eight groups consisting of both breeds and the four diets.

LEfSe-analysis revealed the 25 most significant discriminant ASVs for breed and diet based on the average abundance across the factors combination (breed x diet). Even if no significance for those ASVs was revealed by comparing the dietary groups within the breeds, the average relative abundance changes across the breed x diet combinations. Eleven ASVs were assigned to a species (*Lactobacillus kitasatonis, Ligilactobacillus aviarius, Lactobacillus helveticus, Ligilactobacillus agilis, Megamonas funiformis, Bifidobacterium longum, Sutterella timonensis* and *Negativibacillus massiliensis*) and additional eight were assigned to a genus, the rest remained unclassified at lower taxonomic levels (Figure 5)*.* Additionally, two ASVs belong to the core microbiota (ASV62, ASV197) and were more abundant in LSL compared to LB. Bacterial shifts were revealed across diets for each breed, either increasing or decreasing abundance and between the breeds, where some ASVs show higher relative abundance in one breed compared to the other. These results showed that the breed is the primary driver of microbial composition, followed by the GIT section and Ca/P supplementation.

## Discussion

GIT microbiota in poultry is influenced by many exo- and endogenous factors such as animal age, stress, genotype, or diet (Wickramasuriya et al., 2022). Whereas the microbiome in broilers is extensively researched, knowledge about laying hens is scarce, especially the microbiota description along the whole GIT. Microbiota stimulates the immune system, contributes to host nutrition and pathogen inhibition, synthesizes amino acids and vitamins, and has a role in breaking down complex molecules and potential toxic feed components (Borda-Molina et al., 2016). Changes in microbiota composition, either by feed, disease or other external factors, can affect these functions; thus, its understanding and characterization are of primary importance. Therefore, this study aimed to identify differences in the active microbiota composition along the GIT including digesta and mucosa in two commercial breeds of laying hens fed diets with dietary Ca and P concentrations 20% below the recommended levels.

Among the factors studied in the present work, the breed had the most significant effect on the microbial community, leading to fluctuations in relative abundance on every taxonomic level across the complete GIT. Consistently, breed disparities have been reported in caecal samples of a recent study comparing Hy-Line W36 and Hy-Line Brown (Adhikari et al., 2020). Depending on the diet, such breed-related changes might be due to differences in body weight and average daily feed intake between breeds. Moreover, both breeds have different mechanisms regarding P absorption (Abudabos, 2012) and the significantly higher concentrations of inositol-6 phosphate and inositol-5 phosphate in LB gizzard and caeca (Sommerfeld et al., 2020) might be due to breed-dependent impacts of P, which results in changes in the GIT microbial community.

Previous studies have only characterized the microbiota of single sections of the GIT or feces and showed similar results at phylum and genus levels, as reported here (Stanley et al., 2012; Simon et al., 2016; Ding et al., 2017; Adhikari et al., 2020; Schreuder et al., 2020; Khan and Chousalkar, 2021; Su et al., 2021; Xiao et al., 2021). The use of different breeds also didn’t affect the overall picture of the microbiota, being the main bacterial groups detected across all studied breeds (Ding et al., 2017; Huang et al., 2019; van der Eijk et al., 2019). There is still a discussion on whether richness in microbiome composition is positively (Stanley et al., 2012; Stanley et al., 2014; Yan et al., 2017) or negatively (Siegerstetter et al., 2017) correlated to animal health. The present study found the highest diversity in the caeca, followed by the duodenum and ileum, with statistical differences between breeds. The highest diversity in caeca is consistent with previous studies (Borda-Molina et al., 2016; Fu et al., 2018).

Besides the differences in diversity index, the animal breed affected phyla abundance and species distribution, which was previously reported in broilers (Paul et al., 2021). We detected fewer Firmicutes and higher levels of Bacteroidetes in LB than in LSL. Khan et al. (2021) reported that a lower abundance of Firmicutes in laying hens is associated with a decrease in certain bacteria, including *Peptostreptococcus* (Khan and Chousalkar, 2021) which is contrary to the recent study, where LB with lower abundances of Firmicutes compared to LSL showed no decrease in *Peptostreptococcus*. On the other hand, Bacteroidetes was significantly higher in LB and an increased abundance of Bacteroidetes has been associated with later stages of the laying phase, where the abundance of Firmicutes decreases and Bacteroidetes overtakes (Joat et al., 2021).

One of our aims was to identify the effect of lower supplementation of Ca and P in the GIT, because an insufficient supply of one or both minerals might reduce animal growth and bone mineralization due to interference with homeostasis (Shafey et al., 1990) and change the microbial community of the laying hens. Members of *Ligilactobacillus*, *Megasphaera*, Lachnospiraceae, Bacteroides, *Helicobacter*, Prevotellaceae, *Lachnoclostridium, Streptococcus* and Lactobacillaceae were affected by the diets. The relative abundance of Lachnospiraceae decreased with Ca supplementation, which might have a negative impact to gut health as members of Lachnospiraceae are related to the production of butyrate, crucial for the metabolism of the epithelial tissue (Biddle et al., 2013). The genus *Megasphaera* is known to be part of the SCFA production in the caeca of laying hens (Gan et al., 2020). In our study, the higher Ca supplementation was causing a decrease in this genus’s abundance and might have reduced the SCFA production in LSL. *Ligilactobacillus* and other members of the family Lactobacillaceae are known colonizers of the GIT of laying hens (Forte et al., 2018). In this study, their prevalence changed depending on Ca and P supplementation, breed and GIT section. Members of these genera are usually associated with improved GIT health, productive performance and regulators of the immune system (de Cesare et al., 2017; Forte et al., 2018). In addition, *Streptococcus* is closely related to productive performance with negative correlations to feed conversion ratio (Gan et al., 2020). Higher levels of ASVs belonging to this genus were observed in LB hens supplemented with higher Ca levels and that had probably led to the reduced average daily feed intake under the same conditions in this breed (Sommerfeld et al., 2020). Moreover, in a companion study that used the same hens, P^-^ affected the immune system by increasing immune cell numbers and mitogen-induced response of innate and adaptive immune cells (Hofmann et al., 2021). In contrast, the relative abundance of potential pathogen *Helicobacter* increased with higher levels of P in the diet, which could have indicated some effect on the immune system (Fox, 1997; Miao et al., 2020); however, the numbers of T cells and CD4^+^ increased in the same hens (Hofmann et al., 2021).

Most of the top 25 discriminant ASVs had higher relative abundances in LB compared to LSL, depending on the feature and the fed diet. Finally, the impact of the diet on the microbial composition showed that the offered diets were not challenging the laying hens GIT microbiota. Jing et al. (2018) reported that a reduction to 0.15% available P in the feed was not affecting growth, productive performance, and mRNA expression of P transporters in hens. It was assumed that a lower P and Ca supplementation might lead to functional shifts, as this was observed in a study with probiotic supplementation compared to a standard diet (Iqbal et al., 2021). But, the predicted functional pathways revealed no overall direct influence of P and Ca in the present study.

Previous studies in layers revealed that members of Lactobacillaceae, Bacteroidaceae, Lachnospiraceae, Ruminococcaceae, Veilonellaceae, Prevotellaceae, Clostridiaceae, Rickenellaceae, or Enterobacteriaceae account for the core microbiota (Videnska et al., 2014; Ngunjiri et al., 2019). However, none of the studies combined the information across the complete GIT or targeted the active microbiota. In the present study, five core bacteria were detected across 97% of the samples; uncl. *Lactobacillus*, *Megamonas funiformis*, *Ligilactobacillus salivarius*, *Lactobacillus helveticus* and uncl. *Fusicatenibacter*. Considering the high number of samples (*n* = 678) and the microbiota variation across the GIT, with common colonizers appearing or not in each GIT section digesta and mucosa, the likelihood of finding a core microbiota across all samples decreases (Johnson et al., 2018; Lee et al., 2019; Clavijo et al., 2022). In addition, the detection limit to classify a bacteria as a core member was set to its presence in more than 97% of the total sample number. This percentage is higher than the 50% coverage in Clavijo et al. (2022) and the 75% in Ngunjiri et al. (2019).

All core members are associated with animal health improvement and gut homeostasis. The genus *Lactobacillus* involves host-adapted lactic acid bacteria that colonize the digestive tract of humans and animals (Zheng et al., 2020) and is part of the core microbiome in the ileum and caeca of laying hens (Videnska et al., 2014; Ngunjiri et al., 2019). A beneficial effect on egg size and weight induced by *Lactobacillus* cultures as probiotics was reported (Volf et al., 2021); however, in this study, LSL layers colonized with higher abundances of *Lactobacillus* had lighter egg weights (Sommerfeld et al., 2020). Previous studies have reported *M. funiformis* as a hydrogen consumer in laying hen’s caecal microbiome (Zheng et al., 2020; Volf et al., 2021). It is a characteristic bacterium in adult hens (Volf et al., 2021) and accounted for the core microbiota in a recent broiler study (Clavijo et al., 2022). In our study, *M. funiformis* was found in higher abundance in crop, ileum, duodenum and gizzard samples and almost disappeared in the caeca, which is partially in contrast to the findings of Gan et al. (2020) as they observed the genus *Megamonas* in higher abundances in caeca. The genus *Megamonas* has been previously described in ducks and humans as an important fermenter of glucose into acetate and propionate, which provide health benefits to the host (Chevrot et al., 2008; Sakon et al., 2008). It can be postulated that *M. funiformis* fermented glucose mainly in the upper digestive sections and was displaced in the caeca by other SCFA-producing bacteria*.* Further, *L. salivarius* is commonly isolated from the intestine or faeces of birds and was part of the core microbiome in a recent laying hen study (Ngunjiri et al., 2019). Their response to food-borne pathogens by an antibacterial activity influences the host immune system and the microbial composition (Messaoudi et al., 2013). The LSL hens had a higher abundance of *L. salivarius,* and higher amounts of leukocytes, thrombocytes, monocytes, T cells, T helper cells, and cytotoxic T than LB (Hofmann et al., 2021), which might be a response of the host system to potential pathogens or a breed-dependent reaction to the housing conditions (Moe et al., 2010). *L. helveticus* is an early colonizer of the broiler GIT (de Cesare et al., 2017). Besides the function in pathogen reduction, this bacteria correlated positively with Ca absorption and bone metabolism *in vitro* (Narva et al., 2004). Overall, *L. helveticus* was less abundant in the crop than duodenum and ileum, with main differences between the GIT section of each breed, specifically in LSL. Moreover LSL might be more sensitive to stress, resulting in a more intense immune response and increased blood components (Hofmann et al., 2021) and the potential pathogen reduction and a decrease in stress-induced symptoms can be a breed-related effect. Uncl. *Fusicatenibacter* belongs to the family Lachnospiraceae and was previously associated with host GIT health (Biddle et al., 2013), and detected in the ileum and caeca of laying hens (van der Eijk et al., 2019) with a constant presence from day 1 to week 40 (Asakura et al., 2021). A recent study, using metagenomic analysis, showed several protologues for new candidatus *Fusicatenibacter* (Gilroy et al., 2021), this bacterial group was more abundant in crop and might be involved in the first steps of feed digestion together with *M. funiformis*. The taxonomic core microbiota are microorganisms of a dataset that are postulated to indicate inherent functional relationships with the host. They have the potential to be targeted for culturing and other omics analyses and can be used towards understanding the functional meaning of the core to the laying hen (Neu et al., 2021). The knowledge of the active core microbiota further develops hypotheses about their role within the microbiome.

For the first time, the current study presents data on the active microbiota associated with the whole GIT of two high-yielding laying hen breeds and the core active microorganisms detected in more than 97% of the samples. Significant differences in the microbiota composition were observed between the breeds which was unexpected to such an extent as hens were housed in the same stable, under the same conditions at the same time. Furthermore, we showed that a reduction of circa 20% of Ca and P concentration in the feed compared to the current standard had no effect on microbiota distribution and predicted functions.

## Data Availability

The datasets presented in this study can be found in online repositories. The names of the repository/repositories and accession number(s) can be found below: https://www.ebi.ac.uk/ena, PRJEB52942

## Abbreviations

%, percentage; ASV, amplicon sequencing variant; av. abu, average relative abundance; Ca, calcium; Cae, caeca; Cr, crop; D, duodenum; g, gramm; G, gizzard; GIT, gastrointestinal tract; GS, gastrointestinal section; I, ileum; LB, Lohmann brown-classic; LDA, linear discriminant analysis; LSL, Lohmann LSL-classic; P, phosphorus; Br, breed; SCFA, short-chain fatty acid; uncl, unclassified.
