# Supplementary material for: The active core microbiota of two high-yielding laying hen breeds fed with different levels of calcium and phosphorus
Source: Front Physiol. 2022 Sep 23;13:951350. doi: 10.3389/fphys.2022.951350 (PMC9539745; doi:10.3389/fphys.2022.951350)
Supplement: Supplementary file 2 [file Image1.pdf]

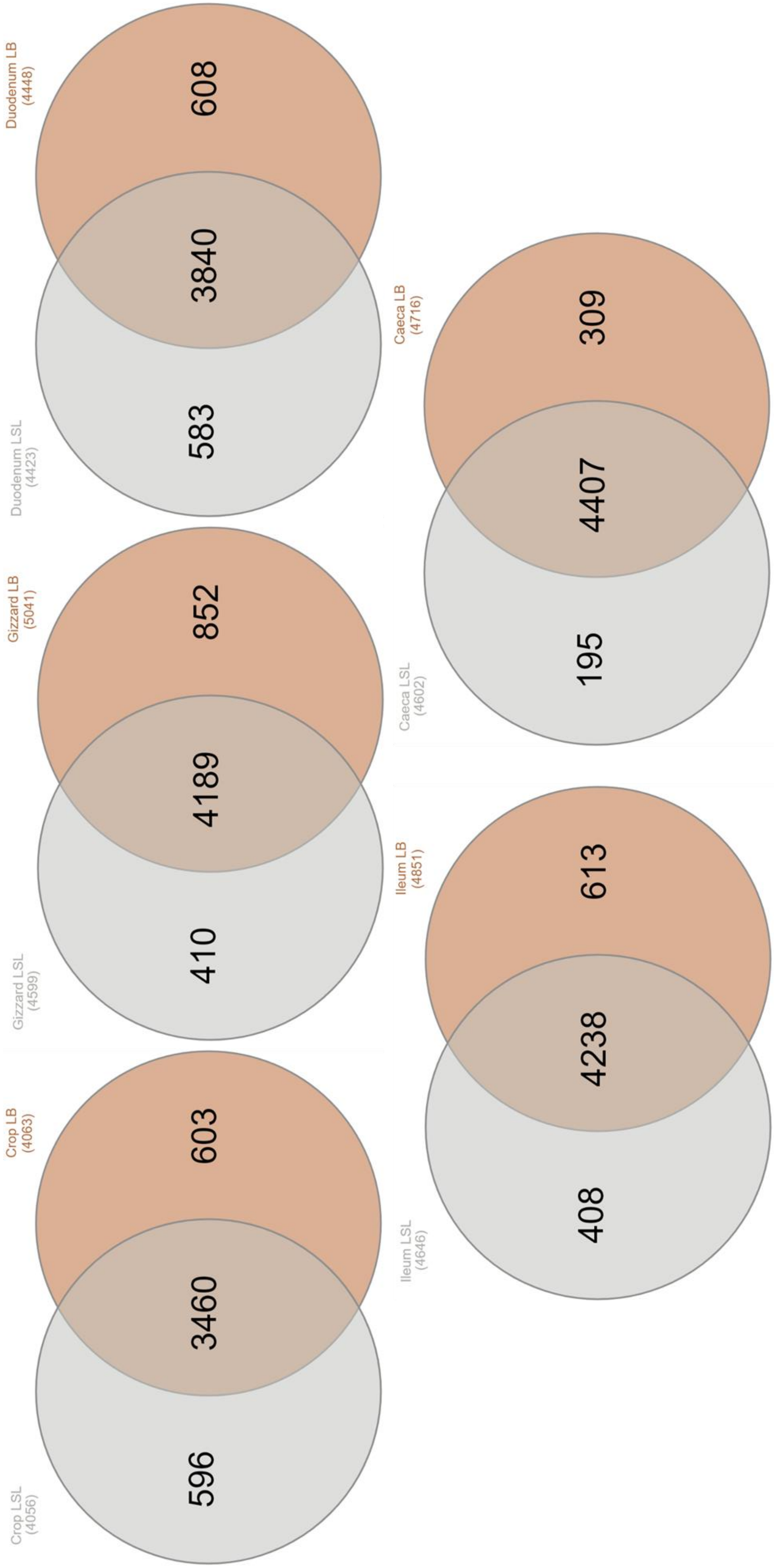

Suppl. figure 1. Distribution of ASV‘s in single GIT sections across all samples in both breeds. The number in parenthesis is the observed number of ASVs in each group.

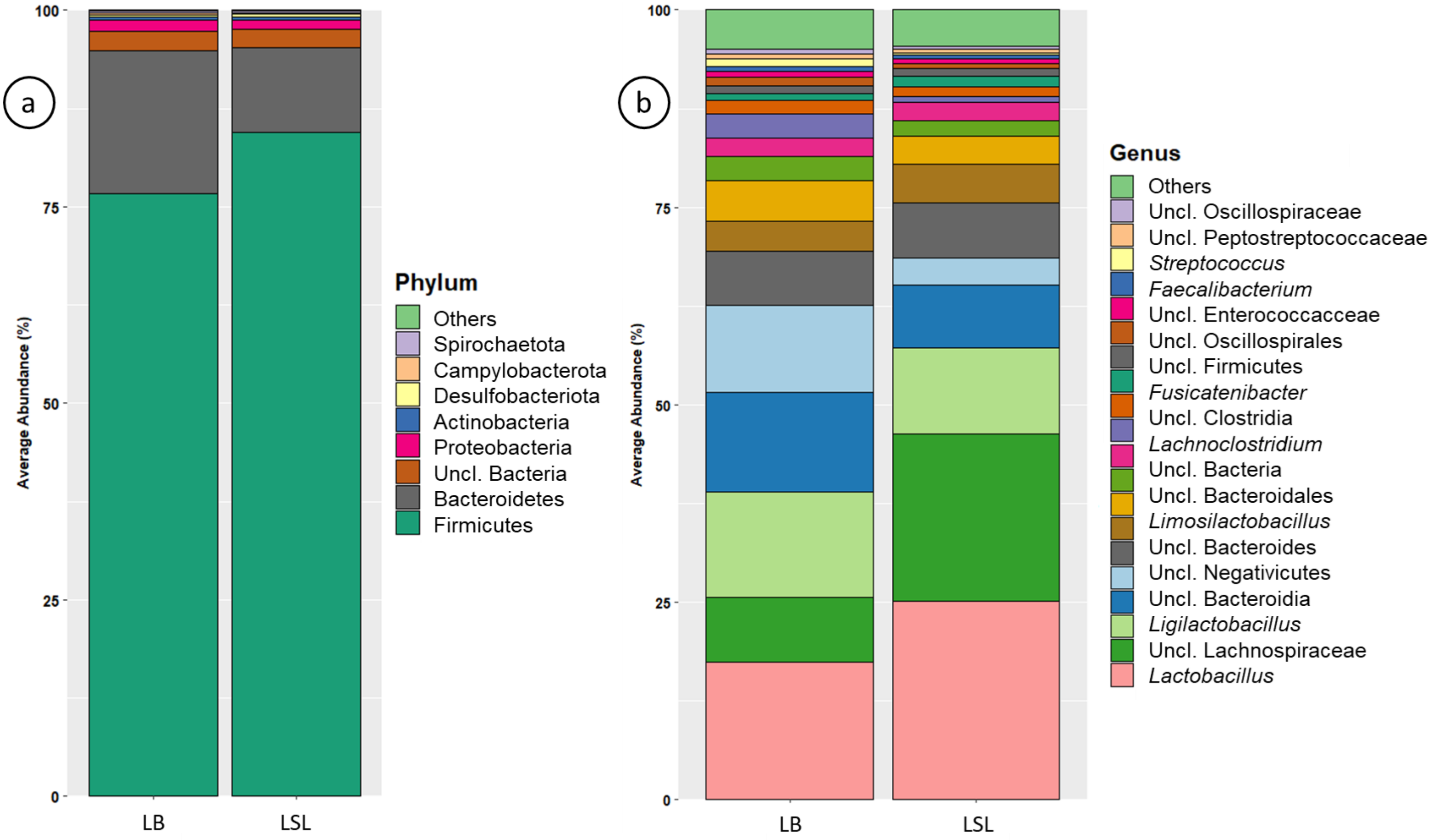

Suppl. figure 2. Barplot of the average relative abundance at phylum level (a) and genus level (b) separated by breed

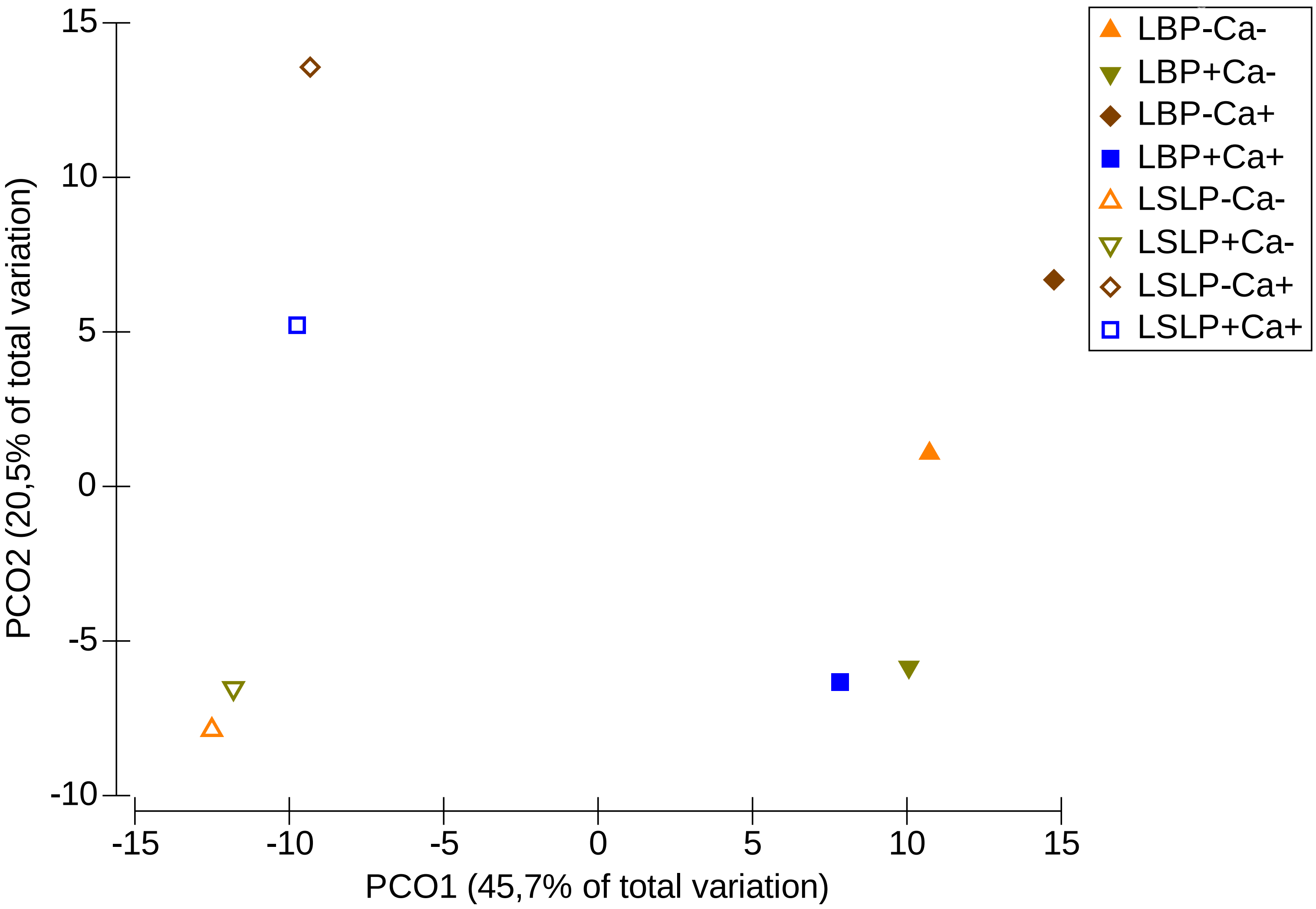

Suppl. figure 3. Centroids of the caecal ASV composition separated by Ca / P supplementation and breed

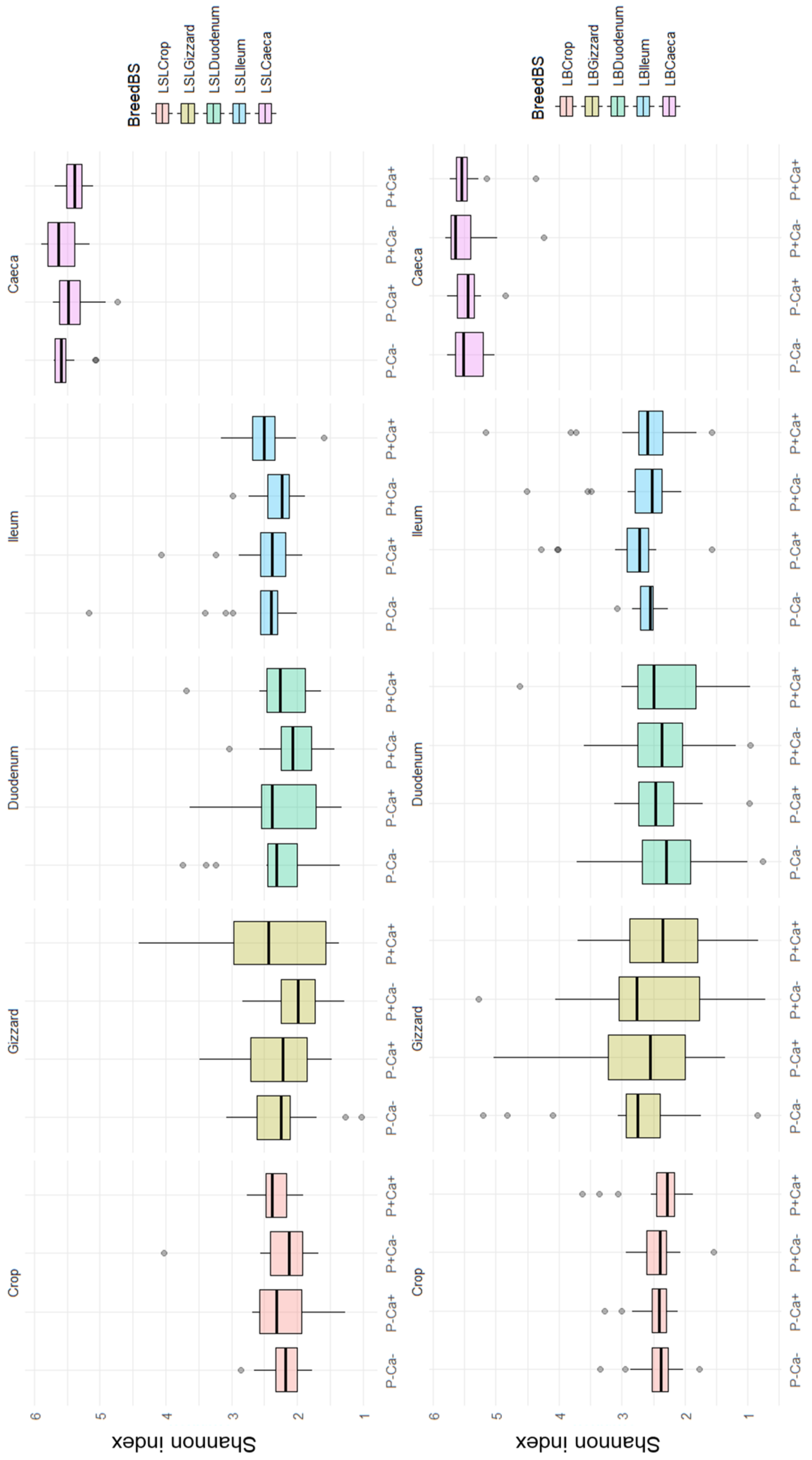

Suppl. figure 4. Boxplot of Shannon diversity index separated by the breed, section (color) and Ca / P combination of the diet

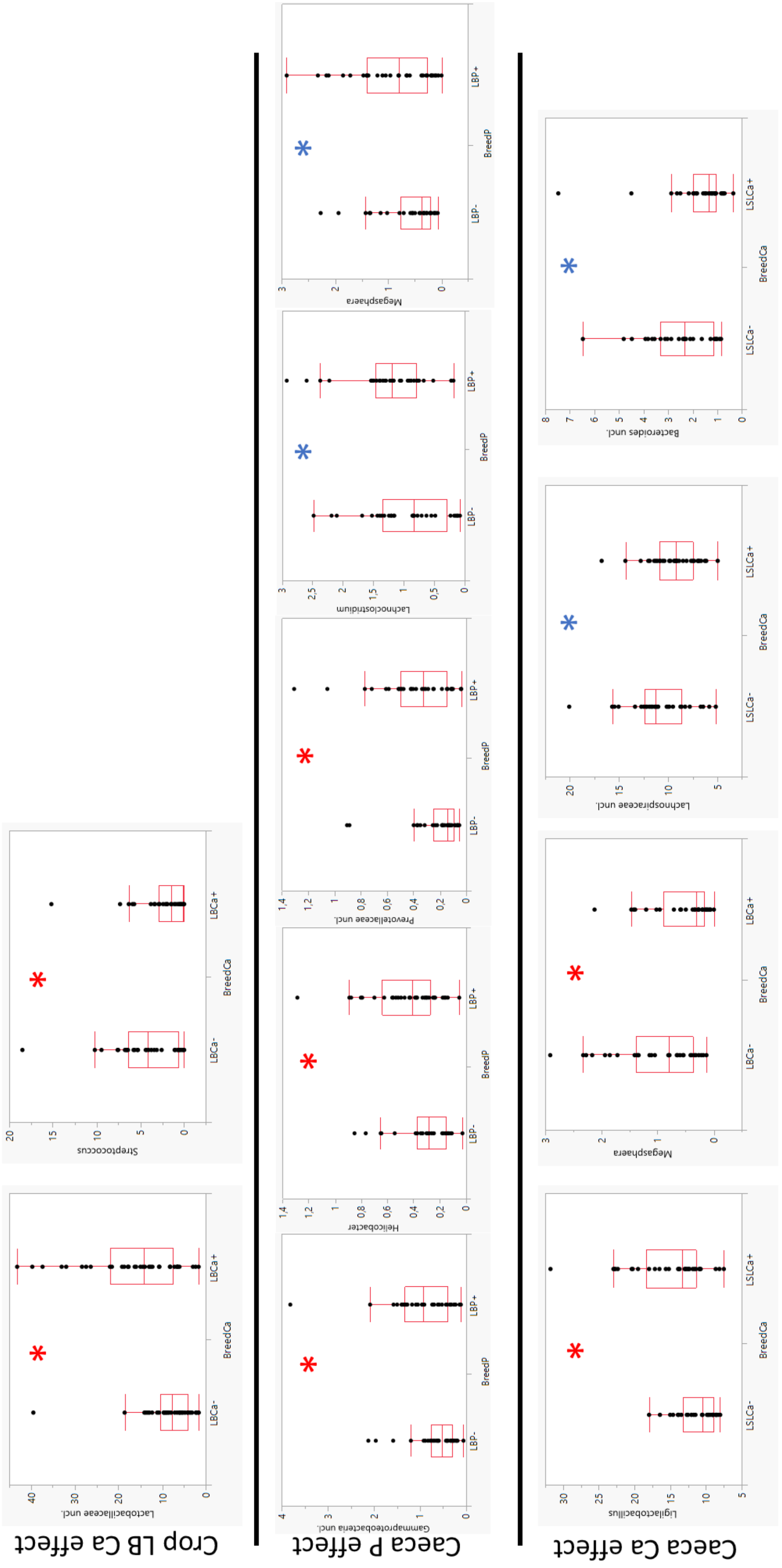

Suppl. fig. 5. Boxplot of the significant (red asterisk) and trending (blue asterisk) changes in crop and caeca related to Ca or P supplementation
